# Retinoic acid is a potential dorsalising signal in the late embryonic chick hindbrain

**DOI:** 10.1186/1471-213X-7-138

**Published:** 2007-12-19

**Authors:** Leigh J Wilson, Anna Myat, Aadhar Sharma, Malcolm Maden, Richard JT Wingate

**Affiliations:** 1 MRC Centre for Developmental Neurobiology, King's College London, 4^th ^floor New Hunt's House, Guy's Campus, London SE1 1UL, UK

## Abstract

**Background:**

Human retinoic acid teratogenesis results in malformations of dorsally derived hindbrain structures such as the cerebellum, noradrenergic hindbrain neurons and the precerebellar system. These structures originate from the rhombic lip and adjacent dorsal precursor pools that border the fourth ventricle roofplate. While retinoic acid synthesis is known to occur in the meninges that blanket the hindbrain, the particular sensitivity of only dorsal structures to disruptions in retinoid signalling is puzzling. We therefore looked for evidence within the neural tube for more spatiotemporally specific signalling pathways using an in situ hybridisation screen of known retinoic acid pathway transcripts.

**Results:**

We find that there are highly restricted domains of retinoic acid synthesis and breakdown within specific hindbrain nuclei as well as the ventricular layer and roofplate. Intriguingly, transcripts of cellular retinoic acid binding protein 1 are always found at the interface between dividing and post-mitotic cells. By contrast to earlier stages of development, domains of synthesis and breakdown in post-mitotic neurons are co-localised. At the rhombic lip, expression of the mRNA for retinoic acid synthesising and catabolising enzymes is spatially highly organised with respect to the *Cath1*-positive precursors of migratory precerebellar neurons.

**Conclusion:**

The late developing hindbrain shows patterns of retinoic acid synthesis and use that are distinct from the well characterised phase of rostrocaudal patterning. Selected post-mitotic populations, such as the locus coeruleus, appear to both make and break down retinoic acid suggesting that a requirement for an autocrine, or at least a highly localised paracrine signalling network, might explain its acute sensitivity to retinoic acid disruption. At the rhombic lip, retinoic acid is likely to act as a dorsalising factor in parallel with other roofplate signalling pathways. While its precise role is unclear, retinoic acid is potentially well placed to regulate temporally determined cell fate decisions within the rhombic lip precursor pool.

## Background

The influence of retinoic acid during early neural development has been extensively characterised [1]. Retinoic acid is required in the formation of the rostrocaudal axis of the neural tube via Hox gene regulation [2,3], as well as in the determination of segmental pattern within the hindbrain [4-8]. Correspondingly, the teratogenic effects of *in utero *exposure to Accutane (13-*cis*-retinoic acid) in humans primarily target the hindbrain [9]: malformations include cystic dilation of the roof of the fourth ventricle, cerebellar vermis dysplasia and/or small cerebellar hemispheres, as well as defects in the inferior medullary olivary and pontine nuclei. Experimental manipulations in mouse, rat and zebrafish show that late-born, noradrenergic nuclei also require retinoic acid for their specification [10,11] and confirm that the cerebellum [12,13] and its major precerebellar afferents, the inferior olive [11,14] and pontine nuclei [15], are particularly sensitive to aberrant retinoid levels. This findings are complemented by observations in the RAREhsplacZ transgenic retinoic acid reporter mouse, which show that the cerebellar vermis, pontine nuclei and inferior olive are all exposed to high levels of retinoic acid signalling during their development [15].

It is a paradox that while retinoic acid has a clear role in early rostrocaudal patterning, it is these late-born, dorsally derived hindbrain components that are particularly sensitive to abnormal retinoic acid signals. The precerebellar system [16,17] and various elements of the cerebellum [18-20] migrate from their birthplace at the rhombic lip, a spatially discrete precursor pool lies at the interface between the dorsal neural tube and non-neuronal roofplate of the fourth ventricle [21,22]. Migratory inferior olive [17] and locus coeruleus neurons [23,24] are born slightly further from the roofplate but adjacent to the rhombic lip. Hence, while retinoic acid has a clear role in specifying the rostrocaudal axis of the neural tube, different lines of evidence point to a separate role in patterning dorsal structures around the fourth ventricle roofplate.

For the post-segmental hindbrain, the meninges have been identified as a potent hypothetical source of retinoic acid [25]. Nevertheless, these tissues, which blanket the neural tube, are unlikely to confer any particular specificity in dorsal signalling. For noradrenergic cells, selective sensitivity appears to reside in their expression of the retinoic acid-induced transcriptional activator AP-2 [10,26]. For precerebellar derivatives, the effects of manipulation of retinoic acid levels have been attributed to a late change in segmental specification of the inferior olive [14] and a failure of tangential migration to the pons [15], inferring two separate roles for retinoic acid within adjacent precursor pools. However, recent results from a transectional genetic study [17] offer an alternative explanation. The developmental consequences of changes in retinoic acid availability for the inferior olive and pontine nucleus are complementary: deprivation causes an increase in size in the former and a decrease in the latter. This phenotype bears a remarkable similarity to that seen in the Pax6 *smalleye *mutant [17,27]. The inferior olive and the pontine nuclei arise at the same rostrocaudal location at different distances from the roofplate. The Pax6 *sey/sey *phenotype has been attributed to a disruption in dorsoventral patterning underlying the allocation of these precursor pools. The effects of retinoic acid deprivation hence mimic a dorsoventral patterning phenotype.

These observations and the teratogenic phenotype of retinoic acid raise the possibility of a more spatiotemporally defined system of dorsal retinoic acid signalling. Consequently, we undertook a survey of the expression of transcripts of members of the retinoic acid signalling pathway. Our results suggest patterning roles for retinoic acid that are distinct from earlier hindbrain axial patterning. Specific nuclear clusters in the hindbrain and mid/hindbrain isthmic region express RNA transcripts for both synthetic and catabolic enzymes indicating hotspots of retinoic acid manufacture and usage. At the rhombic lip, microanatomical spatial expression domains suggest that roofplate-derived retinoic acid might act specifically on rhombic lip precursors as a diffusible, roofplate-derived dorsalising factor.

## Results

We processed embryos aged between embryonic (e) day 3 (stage 20, [28]) and e10 (stage 36) for in situ hybridisation to assess the spatial distribution of mRNA transcripts of participants in the RA-signalling pathway in the developing hindbrain: synthetic enzymes, binding and signalling proteins, and breakdown enzymes. During these stages, different cohorts of rhombic lip derivatives become specified and undergo tangential migration through the cerebellum and hindbrain [29,30].

### Retinoic acid synthesis

**Figure 1:**
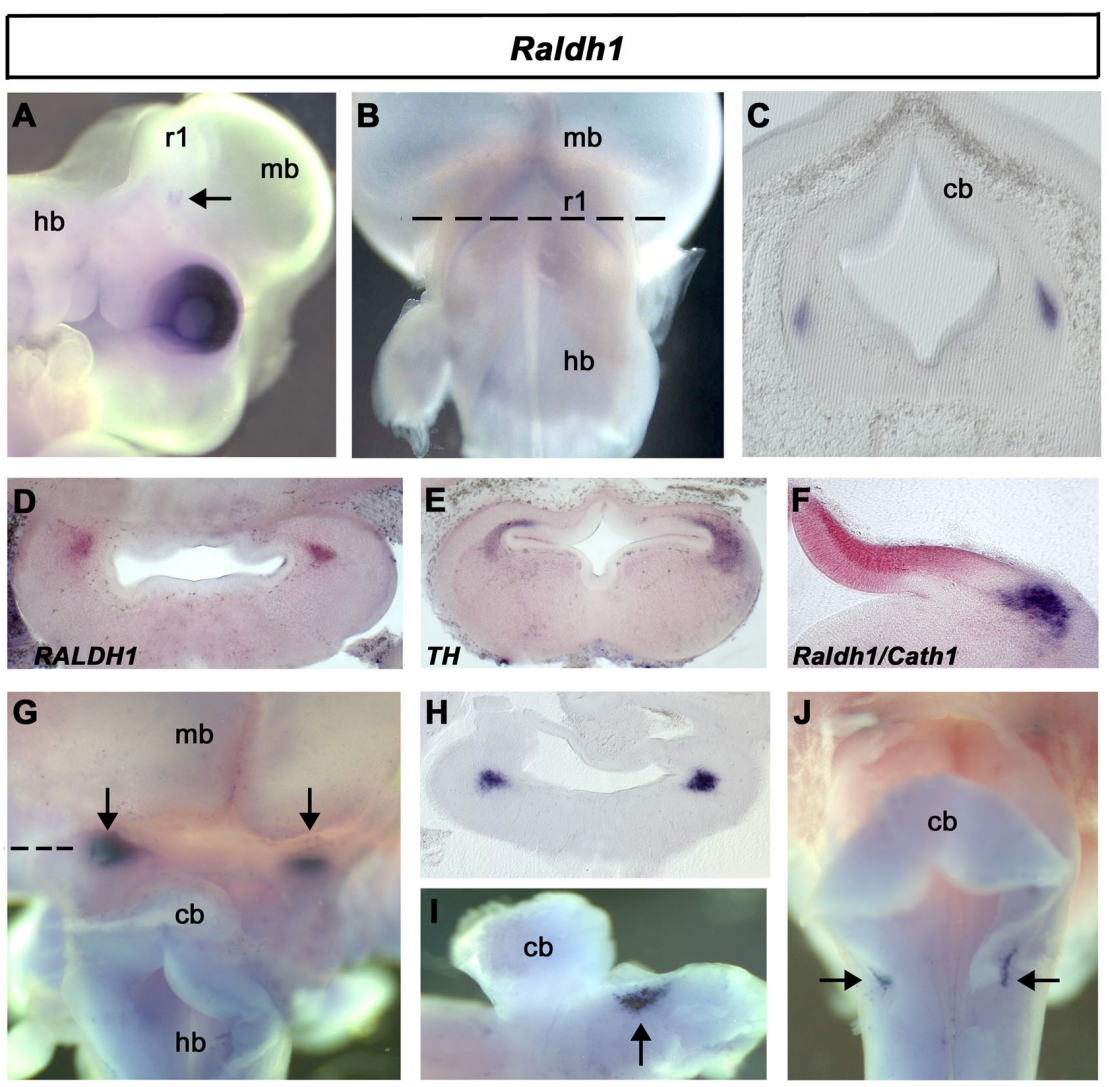
**Expression of *Raldh1***. In situ hybridisation was performed on wholemount embryos with DIG- or fluorescein-labelled riboprobes. Unless otherwise stated, rostral is top, in views of the dorsal embryo, and to the right in lateral views. Abbreviations: midbrain (mb), hindbrain (hb), cerebellum (cb), rhombomere 1 (r1). **A**. At e3.5, in a lateral view of a wholemount embryo, *Raldh1 *is located in the eye and presumptive locus coeruleus (LC) of ventral r1 (arrow). **B**. Dorsal view at e5. **C**. Transverse section (through level indicated by dashed line in B) identifies expression in the LC. **D**. Expression at e6 of *Raldh1 *in a transverse section through rostral rhombomere 1. **E**. Expression of *tyrosine hydroxylase *(*TH*) at e6 in a matched transverse section of rostral r1. **F**. Transverse section through r1 at e6 showing double in situ hybridisation for *Raldh1 *(blue) and *Cath1 *(red). **G**. Dorsal view of hindbrain at e6.5 showing bilateral expression within putative isthmo-optic territory (arrows). **H**. Transverse section through the mid/hindbrain (dashed line in G). **I**. Expression relative to the cerebellum (midbrain removed) in a lateral view at e7 (arrow). **J**. Expression in vestibuloacoustic territory at e7 (arrows).

The production of retinoic acid in the embryo relies chiefly on the action of three retinaldehyde dehydrogenase enzymes (Raldh1, 2 and 3), which perform the second step of the conversion of retinol into the biologically active all-*trans*- and *cis*-retinoic acid. At e3.5, *Raldh1 *is restricted to the dorsal retina and a cluster of cells within rhombomere 1 (Fig. 1A). Transverse sections of rhombomere 1 at e5 (Fig. 1B, C) suggest these cells lie within the noradrenergic, locus coeruleus. To confirm their identity, we examined the expression of mRNA for tyrosine hydroxylase (TH), which converts tyrosine to L-dopa in the synthesis of noradrenalin. In situ hybridisations were performed on stage-matched embryos with *Raldh1 *(Fig. 1D) and *TH *(Fig. 1E) and show that transcripts are co-localised. Double in situ hybridisation reveals that *Raldh1*-positive neurons (blue) lie well outside the developing external granule cell layer (EGL) identified by *Cath1 *(red) expression (Fig. 1F). From ~e6.5, *Raldh1 *expression is found additionally within a discrete bilateral nucleus, rostral to the cerebellum, at the interface between hindbrain and midbrain (Fig. 1G). In transverse section (Fig. 1H) and following more extensive dissection (Fig. 1I), these neurons lie close to the isthmo-optic nucleus [31-33]. At e7, superficial bilateral patches of *Raldh1 *expression can be seen in the vestibuloacoustic region of the hindbrain (Fig. 1J).

**Figure 2:**
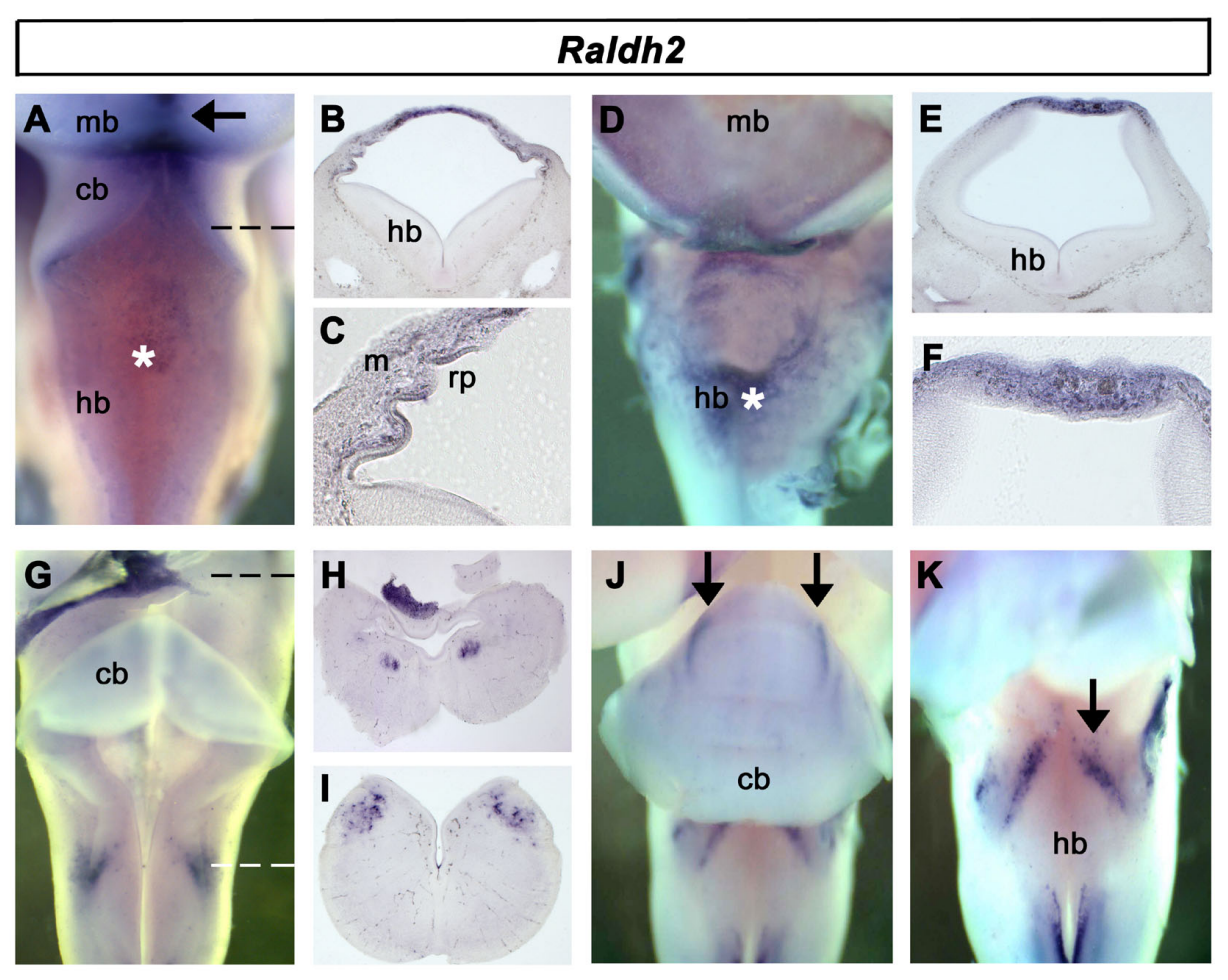
**Expression of *Raldh2***. **A**. *Raldh2 *is expressed in the membranes overlying the IVth ventricle (*) and dorsal midbrain (arrow) at e3.5. **B**. Transverse section through rostral hindbrain (dashed line in A) showing roof plate expression. **C**. Higher magnification of *Raldh2 *expression (in B) shows *Raldh2 *in both the mesenchyme (m) and roofplate (rp). **D**. Expression in the meningeal membranes surrounding the neural tube from e5 (*). **E**. Transverse section through hindbrain shows expression is concentrated in the meninges. **F**. Higher magnification of *Raldh2 *expression in B shows expression through all layers overlying the ventricle. **G**. Whole embryo at e6.5 with meninges largely removed. **H**. Transverse section through midbrain (at level indicated by black dashed line in G) shows expression in the oculomotor nucleus. **I**. Transverse section through caudal hindbrain (at level indicated by white dashed line in G) shows expression in a dorsal neural population. **J**. Dorsal view of e8 whole embryo with meninges removed shows *Raldh2 *in restricted parasagittal domains (arrows). **K**. Dorsal view (same embryo as J) with the cerebellum displaced to reveal dorsal hindbrain expression (arrow).

*Raldh2 *is located at e3.5 in the dorsal midline of the midbrain and the membranes covering the fourth ventricle (Fig. 2A). In section, punctate expression characterises both the ectoderm-derived roofplate and its covering mesodermal membrane, the future dura mater (Fig. 2B, C). From E5, expression in the mesenchyme surrounding the brain becomes more pronounced as the inner leptomeninges begin to condense (Fig. 2D). However, in section it is clear that levels remain relatively elevated in the dorsal mesenchyme (Fig. 2E, F). At e6.5, *Raldh2 *is also expressed in two locations in the neural tube (Fig. 2G): close to or within the oculomotor nuclei (Fig. 2H), and within unidentified neurons of the dorsal, caudal hindbrain, close to the area postrema (Fig. 2I). From e8 onwards, *Raldh2 *expression characterises discrete parasagittal domains within the maturing cerebellum (Fig. 2J) and is maintained in the caudal hindbrain (Fig. 2K).

**Figure 3:**
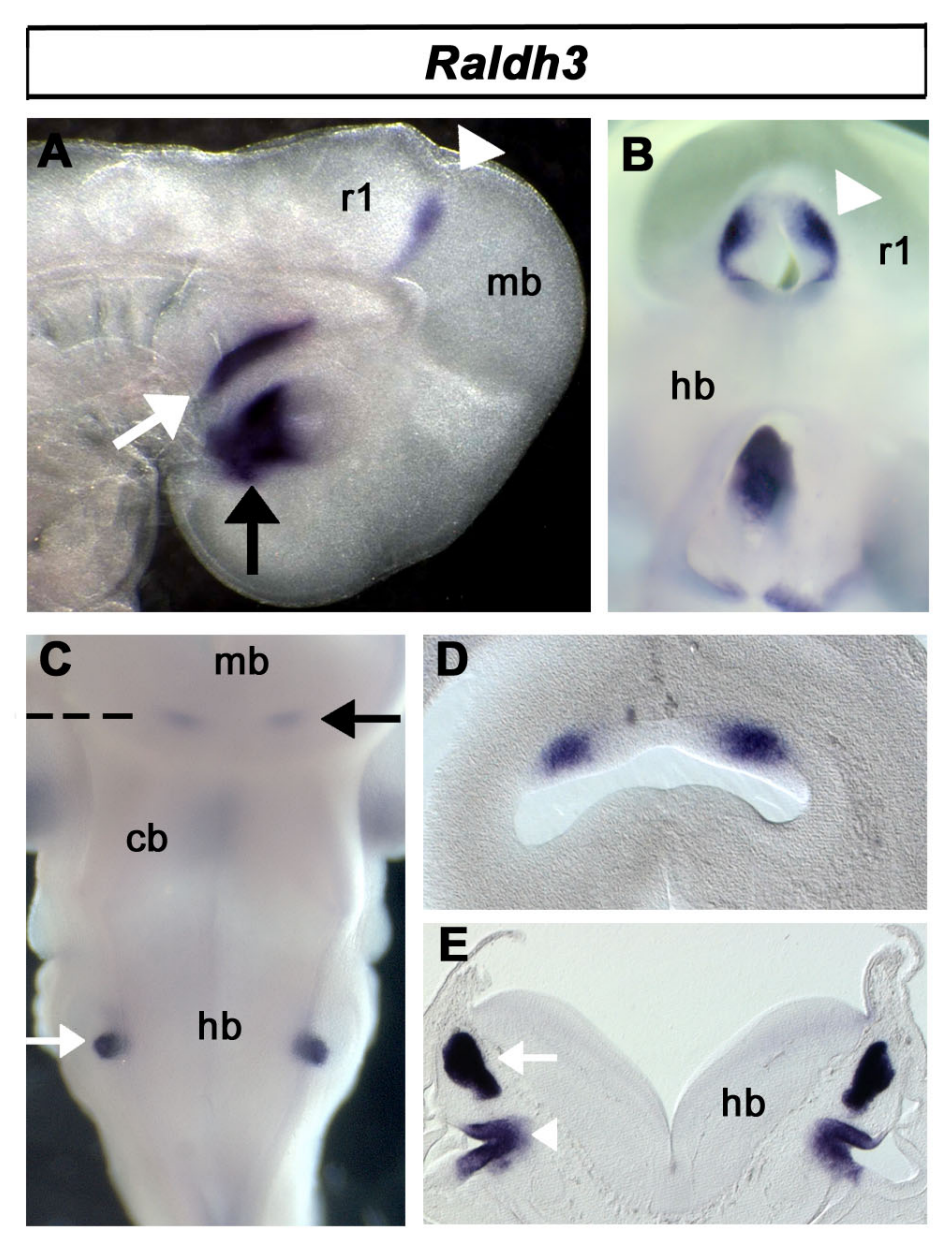
**Expression of *Raldh3***. **A**. *Raldh3 *expression at e3 is localised to the ventral retina of the eye (arrow), Rathke's pouch (white arrow) and the isthmus (white arrowhead). **B**. *En face *view of the dissected isthmus (arrow) showing a ring of *Raldh2 *expression, weakening at its dorsal apex. **C**. Expression at the isthmus (arrow) and otic vesicle (white arrow) at e6. **D**. Transverse section through isthmic region (dashed line in C) shows expression concentrated in bilateral foci within the isthmus. **E**. Transverse section through the presumptive inner ear shows *Raldh2 *in the endolymphatic duct (white arrow) and saccule (white arrowhead).

*Raldh3 *is located at e3 in the ventral retina and Rathke's pouch (part of the presumptive pituitary system [34]). Within the neural tube, as in early embryos [35] expression is localised solely to the mid/hindbrain isthmus (Fig. 3A, B). By e5, this isthmic expression has resolved into two dorsal domains (Fig. 3C, D). The endolymphatic duct and saccule of the inner ear are also significant sites of *Raldh3 *expression (Fig. 3E).

**Figure 4:**
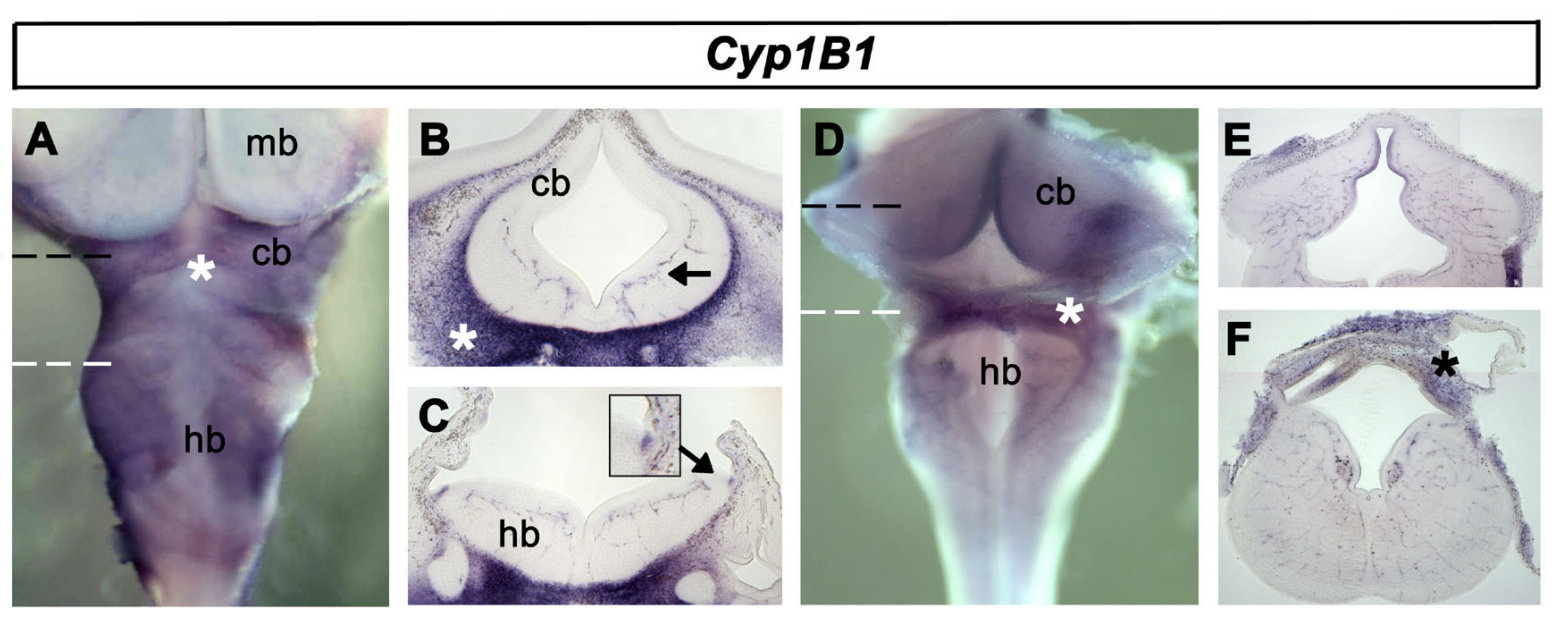
**Expression of *Cyp1B1***. **A**. *Cyp1B1 *expression at e5 is located in the developing meninges (*). **B**. Transverse section through the cerebellar region (black dashed line in A) shows expression in the blood vessels within the neural tube (arrow) and the cranial mesenchyme (*). **C**. Transverse section through the rostral hindbrain (white dashed line in A) reveals expression at the rhombic lip (inset at higher magnification).**D**. Meningeal membrane expression at e7.5 (*). **E**. Transverse section through cerebellum at e7.5 (black dashed line in D) shows meningeal, vascular expression. **F**. Transverse section through e7.5 rostral hindbrain (white dashed line in D) showing expression in the meninges (*) and blood vessels.

To examine the Raldh-independent generation of RA, we also analysed the expression of mRNA transcripts for the cytochrome p450 RA-synthesising enzyme, Cyp1B1 [36]. Expression of *Cyp1B1 *is found in the developing meningeal membranes at e5. In contrast to *Raldh2*, dorsoventral distribution of *Cyp1B1 *is uniform (Fig. 4A). Transverse sections reveal *Cyp1B1 *expression in blood vessels and the adjacent cranial mesoderm (Fig. 4B). *Cyp1B1 *is also expressed at the rhombic lip from e4.5 (Fig. 4C). Expression in the meninges and blood vessels is maintained to e7.5 and beyond (Fig. 4D–F).

### Retinoic acid binding and signalling

Retinoic acid signalling relies on the transport of retinoic acid to the nucleus, where it binds to nuclear receptors to initiate transcriptional control of RA-regulated genes [1]. The availability of retinoic acid to nuclear receptors is regulated by retinoic acid binding proteins (Crabp1 and 2). However, whether Crabp binding potentiates signalling [37-39], or inhibits signalling by sequestering retinoic acid [40] or promoting its degradation [41,42] is subject to debate. Within the nucleus, retinoic acid and RX receptors act heterodimerically as ligand activated transcriptional complexes. We therefore analysed the distribution of a representative binding protein, *Crabp1*, in relation to the mRNA transcripts of three RA-receptors.

**Figure 5:**
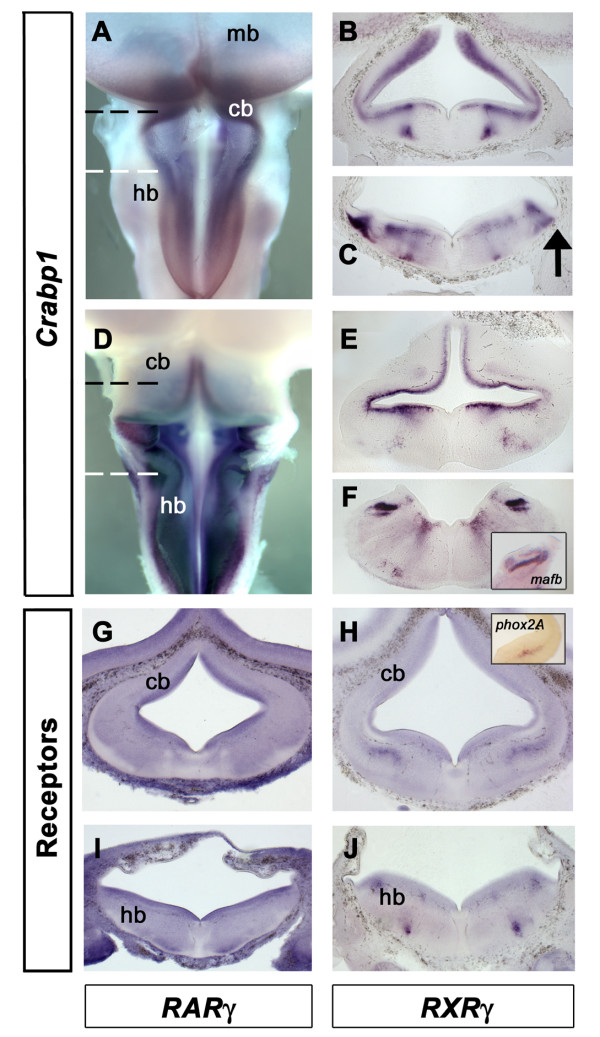
**Expression of retinoic acid binding and signalling components**. **A**. Dorsal view of *Crabp1 *in the rhombic lip at e5. **B**. Transverse section through the cerebellar region (black dashed line in A). *Crabp1 *lies outside the ventricular layer. Dorsally, apparent ventricular expression is a by-product of the plane of section. **C**. Transverse section through the hindbrain (white dashed line in A) with the rhombic lip indicated (arrow).**D**. Dorsal view of an e6 embryo. **E**. Transverse section through the cerebellum (black dashed line in D). **F**. Transverse section through the caudal hindbrain (white dashed line in D) shows expression in the magnocellularis and underlying laminaris of the vestibuloacoustic nuclei, as identified by *mafB *[65] (inset). **G**. *RARγ *expression at the level of the cerebellum at e5 is expressed throughout the neural tube. Areas of lower expression correspond to descending axon tracts. **H**. *RXRγ *expression at the same axial level is ubiquitous but conspicuously elevated in the locus coeruleus, identified by the expression of *TH *or *Phox2a *(inset). **I**. *RARγ *in transverse section is uniformly expressed throughout the neural tube. **J**. In caudal hindbrain, *RXRγ *is elevated in a discrete column of neurons close to the pial surface.

*Crabp1 *expression is widespread throughout the neural tube at e5 (Fig. 5A). Transverse sections reveal that a uniform expression is restricted to the interface between the ventricular layer and the mantle zone (Fig. 5B, C). *Crabp1 *is also strongly expressed in discrete pools of neurons in rhombomere 1, close to the locus coeruleus (Fig. 5B) and within the hindbrain (Fig. 5C). The rhombic lip is surrounded medially by high levels of *Crabp1 *(arrow in Fig. 5C). This pattern is maintained at e6 (Fig. 5D, E). Within the hindbrain, high levels of *Crabp1 *are found in the nucleus magnocellularis and nucleus laminaris of the vestibuloacoustic complex (Fig. 5F).

We examined the expression of RAR and RXR retinoic acid receptors at equivalent stages. Expression of RAR receptors was uniform, widespread and essentially identical between subtypes examined (β and γ). *RXRγ *is widespread throughout the neural tube, while *RXRβ *has yet to be identified in chick. *RXRα *is not expressed in the CNS at these stages [43]. In rhombomere 1, uniform expression of *RAR *(Fig. 4G* RARγ *is shown as representative) contrasts with localised high levels of *RXRγ *expression in the locus coeruleus (Fig. 5H). In the hindbrain, *RARγ *is uniformly expressed in ventricular and mantle zones and absent only from the major descending axon tracts (Fig. 5I). *RXRγ *expression is elevated in the ventricular layer and a small post-mitotic population in caudal hindbrain (Fig. 5J).

### Retinoic acid catabolism

The breakdown of retinoic acid into 4-oxo-RA, 4-OH-RA, 18-OH-RA, and 5, 8-epoxy-retinoic acid occurs via the action of members of the P450 superfamily of Cyp26 enzymes [44-46]. We analysed the expression of the three family members identified so far in chick: *Cyp26A1*, *Cyp26B1 *and *Cyp26C1*.

**Figure 6:**
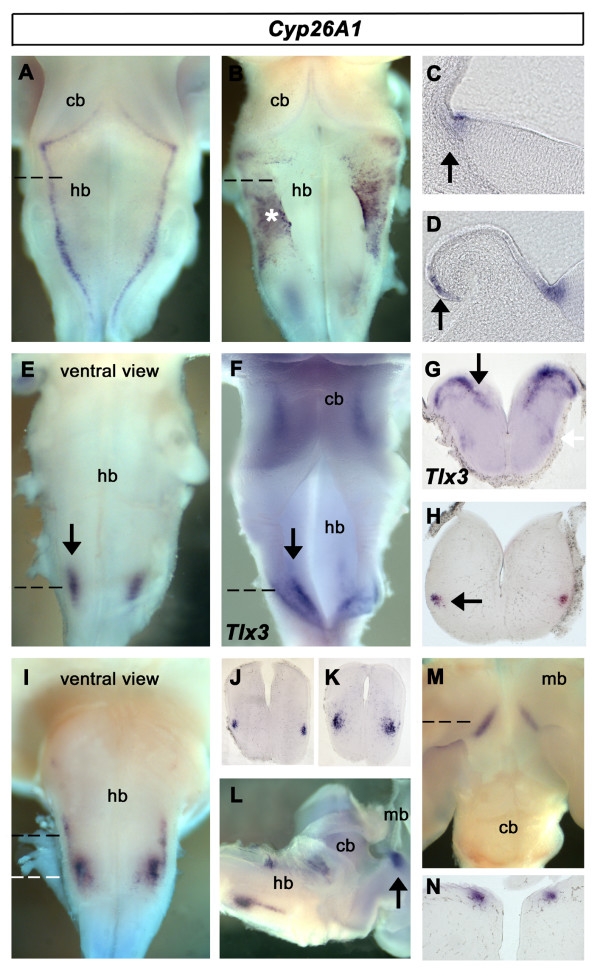
**Expression of *Cyp26A1***. **A**. Dorsal view of *Cyp26A1 *expression in the rhombic lip at e3.5. **B**. Dorsal view of *Cyp26A1 *expression in the rhombic lip and roofplate (*) at e5. **C**. Transverse section through an e3.5 hindbrain (dashed line in A) showing *Cyp26A1 *expression at the rhombic lip (arrow) **D**. Transverse section through an e5 hindbrain (dashed line in B), showing punctate expression of *Cyp26A1 *extending into the roofplate (arrow). **E**. Ventral view of the hindbrain at e5 shows *Cyp26A1 *within a discrete population of neurons. **F**. *Tlx3 *expression at e5 identifies the noradrenergic area postrema (arrow). **G**. In section (dashed line in F), the area postrema (arrow) overlies the nucleus ambiguus (white arrow). **H**. Transverse section through caudal hindbrain (dashed line in E) shows *Cyp26A1 *expression approximately mapping to neurons that express *Tlx3 *(arrow). **I**. Ventral view of hindbrain at e6 shows columnar *Cyp26A1 *expression. **J **and **K**. Transverse sections through rostral and caudal parts of the hindbrain expression domain (dashed black and white lines in I), respectively. **L**. Lateral view of e6 embryo showing relative positions of hindbrain and midbrain label (arrow). **M**. Dorsal view of e8 embryo; discrete expression at the caudal margin of the optic tectum (arrow). **N**. Transverse section through midbrain expression domains (dashed line in M).

*Cyp26A1 *is expressed exclusively at the rhombic lip at e3.5 (Fig. 6A). At e5, punctate *Cyp26A1 *expression is also found in the roofplate into the fourth ventricle (Fig. 6B). The expansion of the *Cyp26A1 *expression domain can be seen in transverse section (compare Fig. 6C and 6D). *Cyp26A1 *also becomes upregulated in a caudal hindbrain nucleus close to the ventral midline (Fig. 6E). At this stage, *Tlx3*, identifies noradrenergic-specific cells in the caudal hindbrain [47] (Fig. 6F). In transverse section, *Tlx3 *characterises a band of neurons located close to the ventricular surface in the area postrema and a deeper column of neurons presumably corresponding to the noradrenergic component of the nucleus ambiguus (Fig. 6G). This latter population also expresses *Cyp26A1 *(Fig. 6H). At e6, *Cyp26A1 *expression is down-regulated in the rhombic lip and roof plate. Within the caudal hindbrain (Fig. 6I), a narrow rostral extension of *Cyp26A1 *(Fig. 6J) abuts the domain corresponding to that at e5 (Fig. 6K). *Cyp26A1 *expression can also be detected at e6 within the midbrain (Fig. 6L). Labelled neurons lie at the caudal seam of the optic tectum (Fig. 6M) in a superficial location approximating to the position of the isthmo-optic nucleus (Fig. 6N).

**Figure 7:**
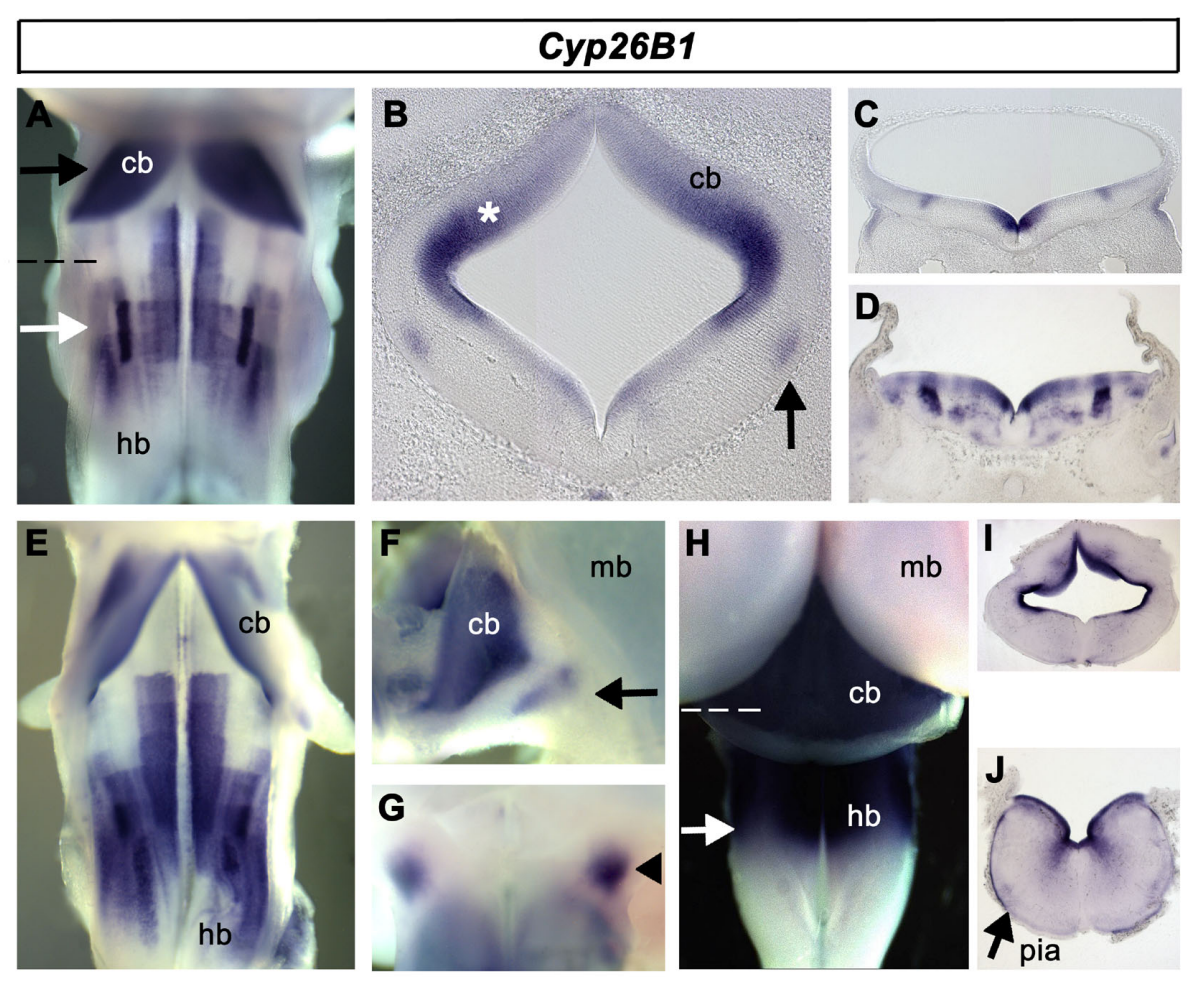
**Expression of *Cyp26B1***.**A**. *Cyp26B1 *expression at e4 in rhombomere 1 (arrow) characterises the cerebellar anlage and a complex patchwork of hindbrain domains, including a dense, lateral column of expression within rhombomeres 5 and 6 (white arrow). **B**. Locus coeruleus (arrow) and ventricular zone (*) in transverse section (at level indicated by black arrow in A). **C**. Transverse section through the caudal hindbrain (indicated by dashed line in A) showing expression in discrete patches of proliferating cells. **D**. Transverse section through rhombomere 5 (white arrow in A) reveals expression in both the ventricular layer and within post-mitotic neuronal populations. **E**. Distinct longitudinal patterns of expression are maintained at e5. **F**. Lateral view of e5 cerebellum and midbrain with locus coeruleus expression (arrow). **G**. Ventral view of mid-hindbrain domain of an e5 embryo showing expression in the region of the isthmo-optic nucleus (arrowhead). **H**. Expression at e7.5 assumes a defined rostrocaudal pattern incorporating the cerebellum, with the caudal limit at the level of rhombomere 6 (white arrow). **I**. Ventricular expression in a transverse section (indicated by dashed white line in H) within the cerebellum. **J**. Transverse section through caudal hindbrain (indicated by white arrow in H) showing expression in ventricular layer and pial membrane (pia, arrow).

*Cyp26B1 *at e4 is uniformly expressed throughout the presumptive cerebellum (Fig. 7A, black arrow). In the hindbrain, *Cyp26B1 *expression reveals a complex patchwork of dorsoventral domains, which display remnants of rhombomeric organisation. *Cyp26B1 *is uniformly expressed in a ventral domain in rhombomeres 2–6. It is highly expressed in a more lateral column in rhombomeres 5 and 6 (Fig. 7A, white arrow). Transverse sections through the neural tube reveal that the majority of *Cyp26B1 *expression lies in the ventricular layer (Fig. 7A–C). Neuronal expression is found in the putative locus coeruleus of rhombomere 1 (Fig. 7B) and the columnar nucleus in rhombomere 5 and 6. This overall pattern of *Cyp26B1 *expression is maintained at e5 (Fig. 7E) and clearly identifies the locus coeruleus, ventrolateral to the cerebellum (Fig. 7F). At this age, discrete domains of *Cyp26B1 *can also be detected at the mid/hindbrain boundary (Fig. 7G) close to the late-appearing domains of both *Raldh1 *(Fig. 1G–I) and *Cyp26A1 *(Fig. 6L). From e6.5, *Cyp26B1 *is uniformly expressed throughout the cerebellum and hindbrain (Fig. 7H). This expression is primarily concentrated in the ventricular zone (Fig. 7I, J) and pial membrane (Fig. 7J), but is not found in overlying meninges.

**Figure 8:**
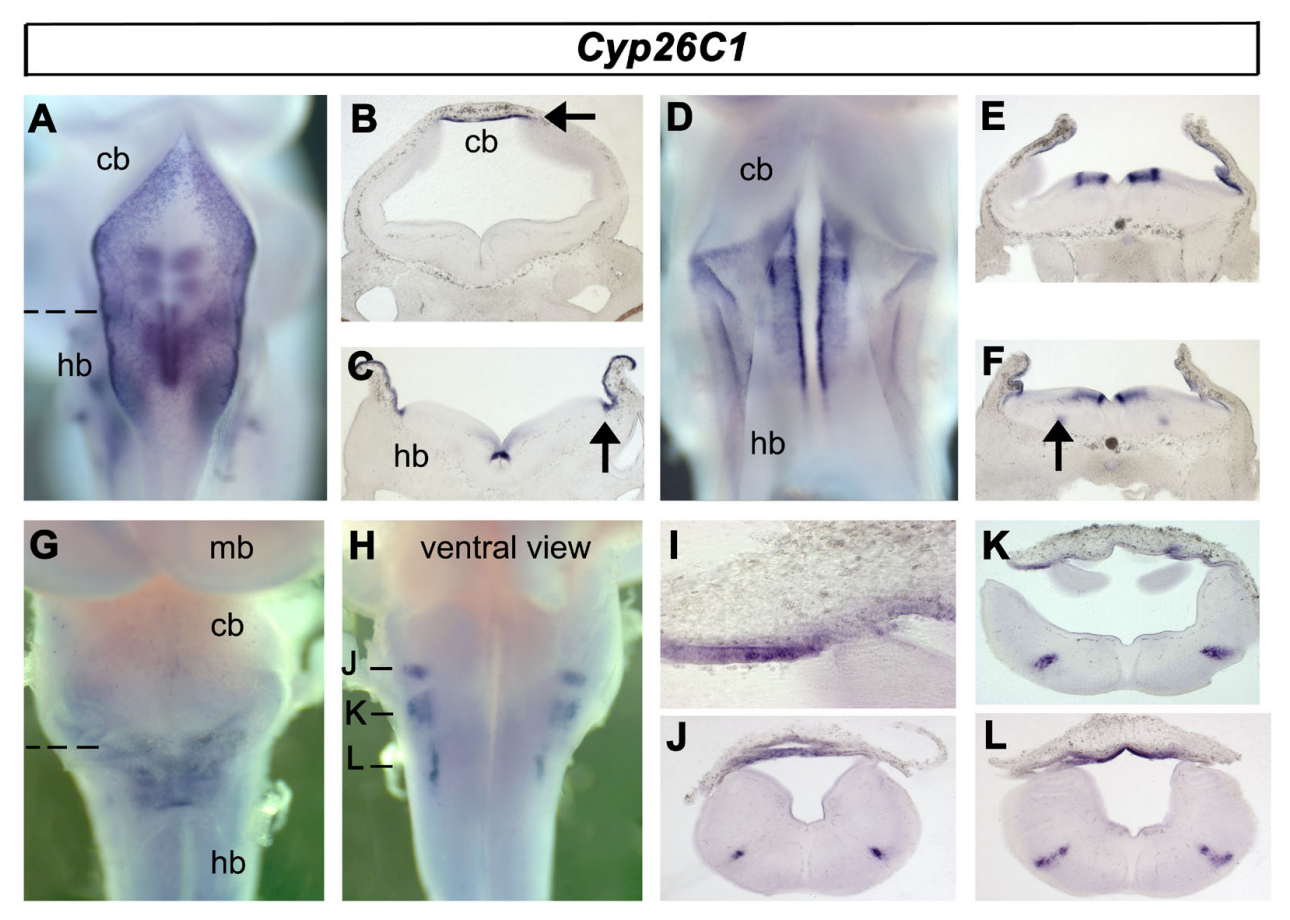
**Expression of *Cyp26C1***. **A**. At e3.5, expression characterises the roof plate, rhombic lip and ventral hindbrain (out of focal plane). **B**. Transverse section through caudal rhombomere 1 showing roof plate expression (arrow). **C**. Transverse section through the caudal hindbrain (dashed line in A) showing expression in the roof plate, rhombic lip and in cells adjacent to the floor plate. **D**. Expression at e5 is similar to that at e3.5. **E**. Transverse section at the level of rhombomere 4 shows that ventral *Cyp26C1 *expression is limited to the ventricular layer. **F**. In addition, at the level of rhombomeres 5 and 6, *Cyp26C1 *characterises a discrete column of post-mitotic neurons (arrow). **G**. Rat e6.5, a dorsal view of the hindbrain shows *Cyp26C1 *is still present in the roofplate. **H**. Ventral view of the same hindbrain shows three ventrolateral *Cyp26C1*-positive populations in the caudal hindbrain. **I**. Transverse section through fourth ventricle roof plate (indicated in G). **J, K, L**. Transverse sections through the caudal hindbrain at positions indicated in H show expression coincident with elevated levels of *RXRγ *(Fig. 5J).

*Cyp26C1 *expression shares characteristics of both *Cyp26A1 *and *Cyp26B1*. At e3.5, *Cyp26C1 *is expressed in the roof plate of the fourth ventricle and at the rhombic lip (Fig. 8A). Ventrally, *Cyp26C1 *is expressed in a segmentally organised ventricular domain. Transverse sections show, as with *Cyp26A1*, that *Cyp26C1 *is expressed only in the roofplate and not the overlying meninges (Fig. 8B). It is most highly expressed at the interfaces between the neural tube and its midline structures, the roofplate and the floorplate (Fig. 8C). This expression is maintained to e5 (Fig. 8D). In transverse section, the ventral ventricular domains of *Cyp26C1 *hindbrain expression (Fig. 8E) are similar to those of *Cyp26B1*. Similarly, in only rhombomeres 5 and 6, *Cyp26C1 *is expressed in a column of post-mitotic neurons (Fig. 8F). At e6.5, expression of *Cyp26C1 *is still present within the roof plate (Fig. 8G) but down-regulated in dividing neural precursors. In the hindbrain, expression is found in three discrete nuclear clusters (Fig. 8H). Transverse sections show that expression of *Cyp26C1 *in the roofplate does not spread to the meninges (Fig. 8I) and that labelled hindbrain nuclei form a contiguous column (Fig. 8K–L) which correspond with higher levels of *RXRγ *(Fig. 5J).

### Retinoid signalling at the rhombic lip

**Figure 9:**
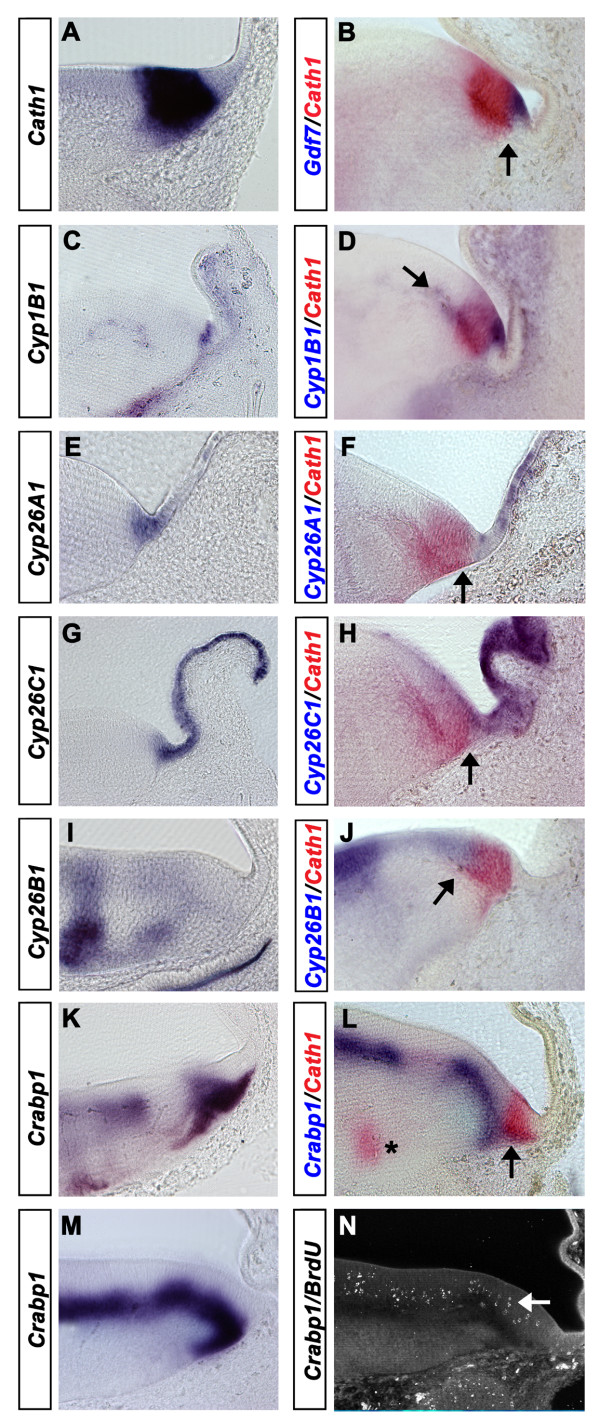
**Retinoid signalling at the rhombic lip**. Transverse sections taken through the caudal hindbrain at e5 with the roof plate orientated to the right and the dorsal neuroepithelium to the left. **A**. *Cath1 *expression at the rhombic lip. **B**. *Cath1 *(red) and *Gdf7 *(in blue) show a non-overlapping interface (arrow). **C**. *Cyp1B1 *expression in dorsal neural tube shows a similar expression to *Gdf7*. **D**. *Cath1 *(red) and *Cyp1B1 *(blue) show no overlap of expression. *Cyp1B1 *is expressed within the developing blood vessels at the base of the ventricular layer (arrow). **E**. *Cyp26A1 *is expressed within the roof plate with highest expression close to the rhombic lip. **F**. *Cath1 *(red) and *Cyp26A1 *(blue) are non-overlapping at their interface (arrow). **G**. *Cyp26C1 *is expressed more uniformly throughout the roofplate than *Cyp26A1 *(E). **H**. *Cath1 *(red) and *Cyp26C1 *(blue) expression is non-overlapping (arrow). **I**. *Cyp26B1 *expression is excluded from the rhombic lip **J**. *Cath1 *(red) and *Cyp26B1 *(blue) show complementary expression domains at the ventricular layer boundary of the rhombic lip. **K**. At the level of the VIIth nerve exit point, *Crabp1 *is expressed at the interface between ventricular zone and mantle layer. **L**. *Cath1 *(red) expression partially overlaps that of *Crabp1 *(blue) at the interface of the ventricular zone (arrow). Transient spots of *Cath1 *expression at the exit point of the VIIth nerve are visible at this level (asterisk). Intriguingly, this post-mitotic expression of *Cath1 *corresponds with a distinct gap in *Crabp1 *at the overlying ventricular layer boundary. **M**. At a slightly more rostral level, *Crabp1 *expression is continuous beneath the ventricular layer. At all levels, there is an extended tail of expression into the mantle layer at the rhombic lip. **N**. Confocal micrograph of BrdU localisation in s-phase nuclei (arrow).

This survey of gene expression indicates a complex pattern of retinoic acid synthesis, transduction and breakdown. Amongst various emergent themes, the spatial domains of transcripts at the roofplate and the rhombic lip suggest that expression boundaries at this interface are tightly regulated. We examined distribution of gene expression of *Cyp *genes and *Crabp1 *at a single time-point (e5) using the basic helix-loop-helix transcription factor, *Atonal1 *(*Cath1*), as a marker of definitive rhombic lip derivatives (Fig. 9A[18,19]) and *Gdf7 *as a marker of the boundary of the non-neuronal roofplate (Fig. 9B[48,49]).

Within the e5 neural tube, the retinoic acid synthetic enzyme *Cyp1B1 *is expressed only at the rhombic lip (Fig. 9C) and within blood vessels. Its expression domain in dorsal neural tube precisely matches that of *Gdf7 *(Fig. 9D) and does not overlap with that of *Cath1*. The retinoic acid breakdown enzymes *Cyp26A1 *(Fig. 9E, F) and *Cyp26C1 *(Fig. 9G, H) share an identical expression pattern which encompasses the roofplate and abuts the rhombic lip. *Cyp26B1 *is widely expressed in the ventricular layer of the neural tube (Fig. 9I). However, as with *Cyp26A1 *and *Cyp26C1*, expression is excluded from the *Cath1*-positive rhombic lip (Fig. 9J). Similarly, transcripts of the retinoic acid binding protein, *Crabp1*, are highly expressed in the region surrounding the rhombic lip (Fig. 9K). The domain of *Crabp1 *precisely abuts the boundary of the *Cath1*-positive precursor domain with a tail of expression extending under the pial surface that matches an extension of *Cath1 *in tangentially migrating cells (Fig. 9L). Injections of BrdU into the neural tube followed by a short (30 minute) survival time reveal s-phase cells adjacent to the layer of *Crabp1 *indicating that *Crabp1 *is expressed in recently post-mitotic cells (Fig. 9M, N). In summary, the various transcripts of retinoic acid pathway proteins are expressed in tissues adjacent to the rhombic lip, but are specifically excluded from the pool of *Cath1*-positive rhombic lip precursors.

## Discussion

**Figure 10:**
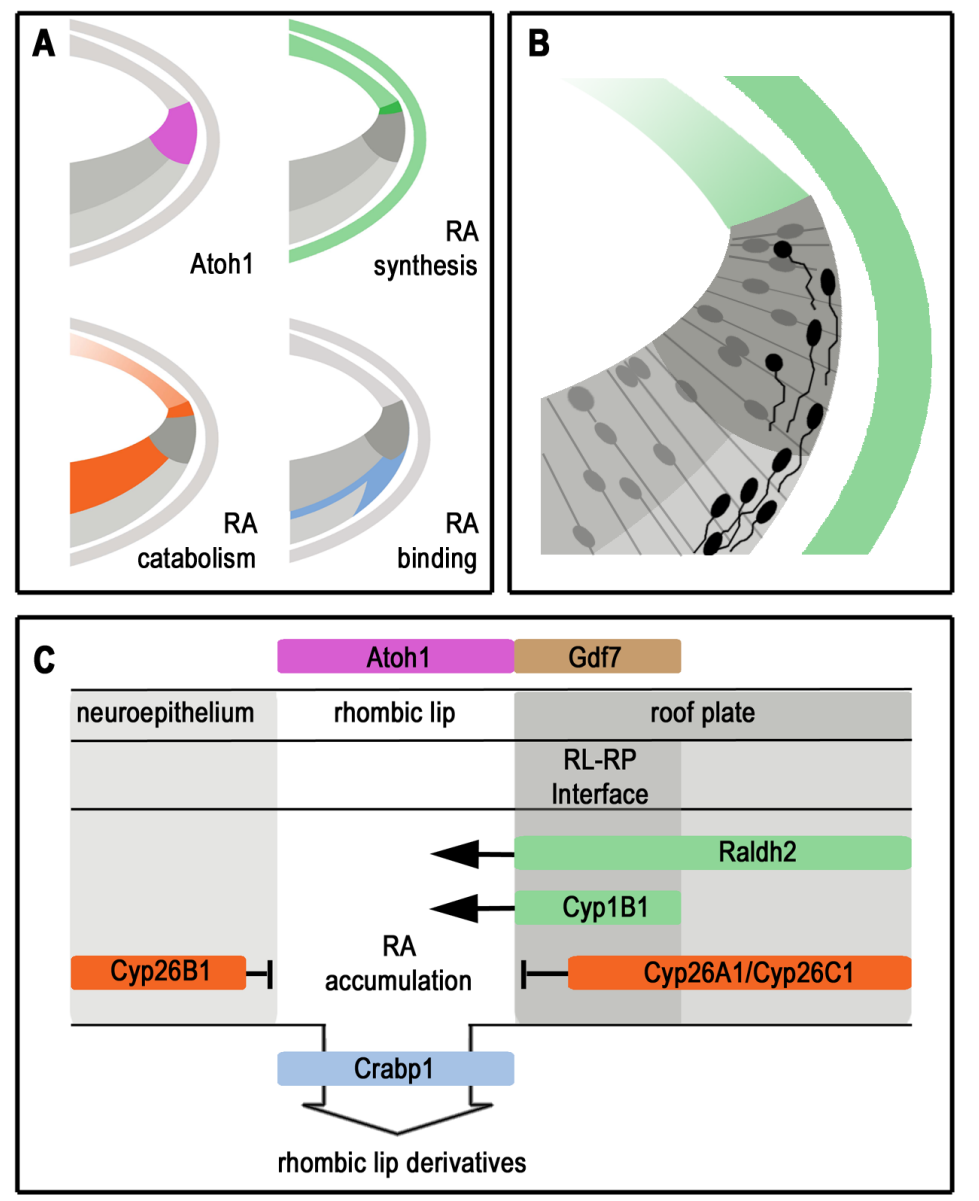
**Summary of results and a model of retinoic acid signalling at the rhombic lip**. **A**. Schematic summary of in situ hybridisation expression domains showing: *Cath1 *(pink) at the rhombic lip; retinoic acid (RA) synthesis (green: *Raldh2 *&*Cyp1B1*) in the roof plate and in the meninges; retinoic acid catabolism (orange: *Cyp26A1*, *Cyp26B1*, and *Cyp26C1*) in the ventricular zone and in the roof plate; retinoic acid binding (blue: *Crabp1*) in the mantle layer. **B**. Cellular events illustrated against the same template as in A, where proliferative cells (grey) and migrating rhombic lip derivatives (black) are shown relative to the production of RA (green) **C**. Model of retinoic acid accumulation resulting from interplay and spatial distribution of retinoic acid signalling components within the dorsal neuroepithelium (left), rhombic lip (middle) and roof plate (right). Expression patterns predict a high concentration of retinoic acid at the rhombic lip. Crabp1 could either play a role in buffering RA or in facilitating its transport to the nucleus as cells exit division. Receptor expression (RAR and RXR) is uniform across the rhombic lip and therefore unlikely to confer signalling specificity.

We have used in situ hybridisation to examine the expression domains of retinoic acid signalling components during late avian hindbrain development. We show that retinoic acid synthesis, catabolism, binding and receptor distribution occur in defined temporal and spatial territories. These observations support a role in the development of specific neuronal populations for retinoic acid signalling that is distinct from its earlier function in patterning the rostrocaudal axis. In contrast to earlier developmental stages, synthetic and catabolic enzymes are co-localised in the same cell groups (Table 1) indicating a highly localised usage of retinoic acid. In particular, detailed characterisation of signalling components at the rhombic lip suggests a role for local retinoic acid signalling at the interface between roofplate and neural tube (Figure 10).

**Table 1:** Retinoic acid signalling components in selected postmitotic hindbrain and isthmic structures

**Retinoic acid pathway component**	non-neural tissue		neural tissue				
		
	**Roofplate**	**Meninges**	**Locus coeruleus**	**Isthmic midbrain**	**Vestibulo-acoustic nuclei**	**Caudal hindbrain nuclei**	
*Raldh1*			√	√^1^	√		synthesis

*Raldh2*	√	√		√^2^		√	

*Raldh3*				√^3^			

*Cyp1B1*	√	√					

*Crabp1*					√		binding

*Cyp26A1*	√			√^1^		√	

*Cyp26B1*			√				breakdown

*Cyp26C1*	√			√^1^		√	

*RAR (βγ)*	ubiquitous						

*RXRγ*	√		√	(√)	(√)	√	

^1^isthmo-optic region, ^2 ^proximal to the oculomotor nuclei, ^3 ^isthmic organiser

### Retinoic acid at the rhombic lip

The rhombic lip is a site of active retinoic acid usage and experimental alterations of retinoic acid levels can profoundly influence production of rhombic lip derivatives [14,15]. However, the assumption that these effects can be traced to late alterations in Hox gene expression and hence axial patterning [15] are confounded by recent studies of Pax6 function at the rhombic lip in the mouse [17]. These analyses infer that the complementary size changes in the inferior olive and pontine nuclei produced by retinoic acid treatment or deprivation might reflect alterations in the dorsoventral allocation of cell fate at the rhombic lip.

'Source and sink' models for the action of retinoic acid based on the distribution of synthetic and catabolic enzymes have been proposed for several systems throughout embryonic development [35,46,50-52]. Figure 10A summarises the results of expression studies of transcripts at the rhombic lip. Transcripts of the retinoic acid synthetic enzymes *Raldh2 *and *Cyp1B1 *are found in the meninges, but also the roofplate and specifically the boundary region between neural and non-neural ectoderm adjacent to the rhombic lip. Breakdown of retinoic acid occurs in the ventricular zone (*Cyp26B1*) and the roofplate (*Cyp26A1 *and *Cyp26B1*) but not within *Cath1*-positive cells. Our results hence indicate that the roofplate is a source for retinoic acid, with potential sinks surrounding, but excluded from, the rhombic lip. Retinoic acid binding protein *Crabp1 *message is found at the interface between dividing cell nuclei and the mantle layer and appears to match the initial stream of tangential migration away from the rhombic lip (Fig. 10B). This suggests that the transcription of *Crabp1 *accompanies exit from the cell cycle. The precise role of Crabp1 in signalling is unclear, either indicating an active transportation and hence utilization of retinoic acid in the nucleus [37-39], or conversely a reduction in the availability of cellular retinoic acid by sequestration [40] or degradation [41,42]. Cells are either activating retinoic acid signalling precisely as they exit the proliferative zone or buffering its further effects (Fig. 10C). An answer to this important question will require a further examination of the role of both *Crabp1 *and *2 *at this transition zone. In either case, our findings correspond with significant activation of *Lacz *in the rhombic lip of the RAREhsplacZ transgenic mouse. We propose that the supply of retinoic acid to the rhombic lip is regulated by a localised gradient from the roofplate.

What are the potential roles of retinoic acid as a diffusible dorsalising factor? Cath1 itself is induced by BMP signals [53,54] downstream of roofplate determinants such as the *Lmx1 *genes [55,56]. It seems unlikely that retinoic acid has a role in inducing *Cath1 *and hence the rhombic lip although our "source/sink" model argues that the primary actions of retinoic acid are within this precursor pool. More consistent with reported functions in other systems would be a function in regulating either the timing of cell cycle exit [57,58] or fate determination [1,59-61]. Cell birth date and fate are intricately linked in the developing rhombic lip with a precise sequence of different populations [29] produced from the same pool of *Atonal1(Cath1)*-positive cells [18,19]. We have recently shown that the transition between temporal fates requires a unidentified signal external to the rhombic lip [62]. Retinoic acid would provide an excellent candidate for such a signal, regulating the progression of cell fate transitions.

If the primary effects of roofplate derived retinoic acid lie within the *Cath1*-positive rhombic lip domain, our model implies that the changes in size of the inferior olive seen with experimental retinoic acid manipulation are either secondary, or via a roofplate-independent source of retinoic acid such as the meninges [25]. The inferior olive precursor pool lies within the dorsal *Wnt1*-positive domain [17] but outside to the *Cath1*-positive rhombic lip [19]. While we cannot exclude a role for the meninges, inferior olive size is likely to reflect both the expression of *Pax6 *at the rhombic lip and mechanisms of cross-repression between proliferating pools. Firstly, retinoic acid within the fourth ventricle roofplate might induce the late, spatially restricted dorsal expression of *Pax6 *at the rhombic lip; loss of *Pax6 *[17,27] appears to mimic the effects of retinoic acid deprivation [14,15]. Alternatively, an increase in inferior olive size may be secondary to disruptions in the rhombic lip. Cross-repression between respective precursor pools within the *Wnt1 *domain has been proposed as a factor that regulates inferior olive size [17].

### Sources and sinks of retinoic acid in late developing hindbrain and isthmic region

Outside the rhombic lip, the proliferative ventricular layer of the hindbrain can be characterized as a patchwork retinoic acid sink by its expression of *Cyp26B1 *in defined blocks of dividing cells. Transcripts of the retinoic acid binding protein, *Crabp1*, characterize the interface between ventricular zone and mantle layer. As at the rhombic lip, *Crabp1 *is likely to be significant in transiently facilitating or terminating intracellular retinoic acid signalling as cells drop out of division.

Outside pools of proliferating cells we find significant "hotspots" of potential retinoic acid signalling in subsets of neurons. Moreover, while in the early embryo the potential sources and sinks of retinoic acid are topographically complementary, there is considerable convergence of expression of retinoic acid pathway transcripts onto a relatively small number of nuclear locations (Table 1). This suggests spatially highly localized or even autocrine retinoic acid signalling. For example, the locus coeruleus is a physiologically significant noradrenergic nucleus with widespread connections throughout the CNS, which is derived from dorsal neural tube [23,24]. Retinoic acid potentiates noradrenalin production by the induction of AP-2, which regulates the transcription of both *tyrosine hydroxylase *and *dopamine beta hydroxylase *[10,26]. In this study, we show that the noradrenergic locus coeruleus is not only a potential sink for retinoic acid but also a source of its production by the expression of *RXRγ/Cyp26B1 *and *Raldh1*, respectively.

Similarly the isthmo-optic region of the caudal midbrain, the vestibuloacoustic nuclei of the hindbrain, and likely noradrenergic nuclei of the caudal hindbrain are both sources and sinks of retinoic acid. Of these, midbrain and vesitbuloacoustic structures have as yet no reported sensitivity to aberrant retinoic acid levels. Finally, a potentially significant source of retinoic acid for both midbrain and anterior cerebellum is the midbrain/hindbrain isthmus. In this case, it is significant that the mammalian cerebellar vermis, which is derived from territory close to the isthmus [63], is both a site of high retinoic acid availability [15] and is specifically affected by exposure to retinoic acid [9,12,13].

The evidence of highly localised signalling within defined postmitotic hindbrain nuclei suggests that retinoic acid is required for the maintenance of these structures. This would explain why specific populations of hindbrain neurons are particularly sensitive to abnormal retinoic acid levels during late development. As with the rhombic lip, this close analysis of retinoic acid pathway transcripts in the remainder of the hindbrain and isthmus reduces a hypothetical requirement for sources of retinoic acid in the meninges [25] or external to the neural tube such as the *Raldh3-*positive inner ear (Fig. 3) and the cranial mesoderm, which expresses *Cyp1B1 *(Fig. 4). The localized sources of retinoic acid production and breakdown that we have identified in this study suggest the possibility of a far more spatially precise signalling system in the late embryonic hindbrain and isthmus.

## Conclusion

The localisation of retinoic acid signalling components in the late developing hindbrain strongly suggests that spatially organised retinoic acid is important in the maintenance of several late-developing neuronal populations such as the locus coeruleus. In addition, we propose that the precise location and spatiotemporal arrangement of retinoic acid pathway transcripts around the rhombic lip and roofplate of the fourth ventricle indicate that retinoic acid is a potential roofplate derived dorsalising factor in the hindbrain (Fig. 10). In this context, retinoic acid is an excellent candidate for regulating the orderly temporal sequence of cell fate changes within the rhombic lip that characterise the production of both its precerebellar and cerebellar derivatives.

## Methods

### Embryo collection and staging

Embryos were incubated at 37°C and harvested in phosphate buffered saline at e3–e10. Dissected neural tissue was fixed in 4% paraformaldehyde and stored at 4°C. Embryos were staged (st.) according to Hamburger and Hamilton [28] along with the corresponding embryonic days of development, such that: e3 = st.20, e4 = st.24, e5 = st.27, e6 = st.29, e7 = st.31, e8 = st.34, e10 = st.36.

### BrdU labelling

BrdU labelling was carried out as previously described (Myat et al., 1996). Embryos were incubated for a 30 minutes following BrdU injection and then fixed in 4% paraformaldehyde

### In situ hybridisation and immunohistochemistry

Tissue was processed for in situ hybridisation using standard protocols [64] with DIG- and fluorescein-labelled riboprobes for a number of genes:, *RARγ, RXRγ *(gift of Paul Brickell), *RARβ *(gift of Gregor Eichele), *Raldh1, Raldh2, Raldh3, Cyp1B1, Cyp26A1, Cyp26B1, Cyp26C1 *[35], *Cath1 *[62], *Tyrosine hydroxylase *[23], *Tlx3 *(gift of Cairine Logan), *Gdf7 *(gift of Anthony Graham), *mafB*, *Phox2a*. At least 15 embryos were processed for each probe and combination of probes in at least three separate experiments. There was no variability in patterns of labelling between experiments. After staining, selected embryos were embedded in 20% gelatin, and sectioned at 40–60 μm on a Leica vibratome. Tissue that had incorporated BrdU was processed with an anti-BrdU monoclonal antibody (Serotec) at 1:40 and an AlexaFluor488 conjugated goat anti-rat secondary antibody (Invitrogen) at 1:50.

### Imaging

Wholemount embryos were digitally imaged on a Leica stereo photomicroscope equipped with epifluorescence. Images of sections were captured using a Zeiss Axiocam fitted to a Zeiss Axiophot microscope. Confocal images were acquired using an Olympus Fluoview laser scanning microscope.

## Authors' contributions

LW, AM and AS performed the in situ hybridisations that form the basis of this study. LW analysed the data and drafted the manuscript. RW, LW and AM finalised the text. LW, RW and MM devised this study. All the authors have read and approved the final manuscript.
